# The Impact of Confinement Configurations on the Compressive Behavior of CFRP—Wrapped Concrete Cylinders

**DOI:** 10.3390/ma18153559

**Published:** 2025-07-29

**Authors:** Riad Babba, Abdellah Douadi, Eyad Alsuhaibani, Laura Moretti, Abdelghani Merdas, Saci Dahmani, Mourad Boutlikht

**Affiliations:** 1 Faculty of Science and Technology, University of Tamanghasset, Tamanghasset 11001, Algeria; riad.babba@univ-tam.dz (R.B.); saci.dahmani@univ-tam.dz (S.D.); 2 Civil Engineering Research Laboratory of Sétif (LRGCS), Department of Civil Engineering, Setif 1 University-Ferhat Abbas, Sétif 19000, Algeria; abdellah.douadi@univ-setif.dz (A.D.);; 3 Emergent Materials Research Unit (EMRU), Setif 1 University-Ferhat Abbas, Sétif 19000, Algeria; abdelghani.merdas@univ-setif.dz; 4 Department of Civil Engineering, College of Engineering, Qassim University, Buraidah 52571, Saudi Arabia; 5 Department of Civil, Building and Environmental Engineering, Sapienza University of Rome, Via Eudossiana 18, 00184 Rome, Italy; laura.moretti@uniroma1.it

**Keywords:** carbon fiber-reinforced polymer (CFRP), confined concrete cylinders, full, partial, and non-uniform confinement, stress–strain behavior, predictive modeling

## Abstract

Experimental tests on confined concrete specimens are essential to characterize the mechanisms activated under varying degrees of confinement. Such characterization is critical for understanding how full, partial, and non-uniform wrapping configurations influence strength and ductility enhancements. This study investigates the compressive behavior of concrete cylinders (160 mm × 320 mm) confined using full, partial, and non-uniform carbon fiber-reinforced polymers (CFRP) configurations. In the first phase, all wrapping schemes were applied with equivalent quantities of CFRP, enabling a direct performance comparison under material parity. The results indicate that non-uniform confinement (NUC) achieved approximately 15% higher axial strength than full confinement (FC2) using the same amount of CFRP. In the second phase, the NUC configuration was tested with 25% less CFRP material, yet the reduction in strength was limited to about 3%, demonstrating its superior efficiency. A new predictive model was developed to estimate peak axial stress and strain in CFRP-confined concrete cylinders. Compared to existing models, the proposed model demonstrated greater predictive accuracy (R^2^ = 0.98 for stress and 0.91 for strain) and reduced error metrics (RMSE and scatter index). ANOVA confirmed the statistical significance of the model’s predictions (*p* < 0.00001 for stress, *p* = 0.002 for strain). These findings highlight the performance advantages and material efficiency of non-uniform CFRP confinement and support the utility of the proposed model as a practical design tool for developing advanced confinement strategies in structural engineering.

## 1. Introduction

The maintenance and rehabilitation of aging or structurally compromised concrete present significant challenges for civil engineers, especially in areas vulnerable to natural disasters, environmental degradation, or design deficiencies [1,2,3]. In recent years, carbon fiber-reinforced polymer (CFRP) composites have emerged as a highly effective solution for strengthening concrete structures due to their high strength-to-weight ratio [4,5], capacity to sustain large deformation [6,7], and corrosion resistance [8,9]. Extensive research has demonstrated the effectiveness of CFRP confinement in enhancing concrete structural performance [10,11]. The mechanical performance of CFRP-confined concrete is considerably influenced by factors such as fiber orientation [12], strip width [13], and number of layers [14,15,16]. Several studies have confirmed that the confinement effect on strength and ductility is more significant in low-strength concrete columns [17,18], and application techniques notably influence the mechanical behavior of CFRP-confined concrete structures [19,20,21,22,23]. However, a persistent challenge in CFRP applications is the discrepancy between the fibers’ ultimate strain and the confined concrete’s failure strain [24]. The CFRP strain efficiency factor (k_ε_), which quantifies this difference, typically ranges from 0.274 to 1.133 [25,26,27,28]. While the strain efficiency factor quantifies the relative increase in strain due to confinement, the mechanism behind this improvement lies in the lateral restraint imposed by CFRP, which enhances the stress state of the concrete and delays failure propagation. This effect is particularly significant in low-strength concrete, where the unconfined strain limit is relatively low. Notably, Lim et al. [29] reported that this efficiency decreases as the strength of unconfined concrete and the modulus of CFRP increase, suggesting a complex interaction between material properties and confinement effectiveness.

While full CFRP confinement is extensively utilized, partial confinement is gaining traction due to its potential to enhance material efficiency and its ease of application. Partial confinement can mitigate debonding [30,31], enhance adhesion by facilitating the escape of trapped air, and simplify the application process [32]. Ismail and al. [33] reported significant strength gains in partially confined elements. Recent advances in fiber-reinforced polymer (FRP) technologies have led to the exploration of various CFRP and glass FRP (GFRP) confinement strategies for improving the axial performance of concrete cylinders [34]. Pham et al. [35] and Siqi Lin et al. [36] demonstrated that non-uniform confinement improves axial strength and strain capacity and enhances material utilization along the cylinder height. Similarly, Junlong Yang et al. [37] and Junlong et al. [38] confirmed that non-uniform CFRP configurations, particularly those with reduced strip spacing or increased layers, offer higher confinement efficiency and delay brittle failure. These findings are supported by Mai et al. [39], who reported that non-uniform wrapping improves the strength and ductility of square reinforced concrete (RC) cylinders and prevents sudden failure; however, full wrapping remains more effective when using identical CFRP quantities. Mai et al. [40] further showed that full uniform wrapping provides superior performance for circularized RC columns compared to non-uniform confinement with the same material.

According to the existing literature [41], selecting between full, partial, and non-uniform confinement strategies largely depends on structural requirements, access limitations, and budget constraints. Nevertheless, systematic comparative studies evaluating these confinement strategies are relatively scarce, creating a knowledge gap concerning the optimal usage of CFRP. This study addresses the gap by systematically evaluating the compressive behavior of concrete cylinders under full, partial, and non-uniform CFRP, using equivalent material quantities.

The characterization of confined concrete through standardized cylinder tests provides essential baseline data for understanding the axial performance of concrete under lateral confinement, particularly when strengthened with FRP systems. These tests yield critical parameters such as confined compressive strength, ductility enhancement, and confinement efficiency, which can be embedded into constitutive models used in the design of structural elements such as short concrete columns. Several confinement-oriented design frameworks, including those proposed by the FIB Model Code and ACI guidelines, incorporate similar empirical formulations derived from cylinder tests to predict load-carrying capacity and ultimate strain. Accordingly, the present study not only contributes to the fundamental understanding of confinement effects but also serves as a foundation for extrapolating these behaviors to the structural scale, particularly in the retrofitting of deficient or aging columns.

The concrete strength level was intentionally selected to represent low-strength or deteriorated concrete, which is frequently encountered in aging infrastructure, particularly in older buildings, bridges, and under-designed structural elements. These types of structures are prime candidates for retrofitting using externally bonded CFRP systems. The results demonstrate that non-uniform confinement layouts can achieve equal or superior structural performance compared to full confinement, even when using less CFRP, thereby offering a more sustainable and cost-effective design alternative.

The essential contribution of this study to materials engineering lies in two aspects: first, it provides experimental evidence supporting the strategic use of discontinuous confinement patterns to improve material efficiency without compromising structural performance; second, it introduces a statistically validated predictive model which is benchmarked against existing models (FIB Bulletin [42], Wang et al. [34]) capable of accurately estimating stress and strain in confined concrete across various wrapping configurations. This dual contribution not only advances the current understanding of CFRP confinement mechanics but also delivers a practical tool for optimizing retrofitting strategies in the field of structural rehabilitation [43]. However, it is important to recognize the inherent limitations of small-scale laboratory tests. These include idealized boundary conditions, uniform stress distribution, and perfect bonding assumptions that may not fully replicate field conditions such as load eccentricity, slenderness effects, or environmental degradation. Therefore, further research involving full-scale specimens, multi-axial loading scenarios, and long-term performance evaluations is necessary to bridge the gap between material characterization and reliable structural design.

## 2. Materials and Methods

### 2.1. Material Properties

The specimens were cast using a standard concrete mix designed to achieve a compressive strength of 20 MPa, calculated using the Dreux–Gorisse method [44]. After 50 days of curing, the average compressive strength was 21.42 MPa, with an ultimate axial strain of 0.408%. This strength level, though moderate, is representative of the mechanical performance typically observed in aging infrastructure, such as older concrete bridges and buildings, making the specimens suitable for evaluating confinement-based retrofitting strategies. Table 1 summarizes the mix proportions and material properties.

The confinement was applied using SikaWrap^®^-230C/45, a unidirectional CFRP fabric, and SikaDur^®^-330, an epoxy resin recommended for FRP applications. Both products were supplied by Sika El Djazair company, the Algerian branch of Sika Group, located in Algiers, Algeria. Table 2 lists properties of the CFRP fabric. 

The mechanical properties of the CFRP composite, consisting of fabric and resin, were determined via coupon testing according to [45]. The results indicate a thickness per layer $t_{f}$ of 1 mm, an elastic modulus $E_{f}$ of 34,900 MPa, a tensile strength $f_{pr}$ of 480 MPa, and an ultimate strain $\varepsilon_{fu}$ of 2%.

### 2.2. Specimen Preparation

Nine series of cylindrical specimens (160 mm diameter × 320 mm height) were tested under axial compression by varying the type of confinement: NC for non-confined specimens, FC for fully confined cylinders, PC for partially confined cylinders, and NUC for non-uniformly confined cylinders. Each series comprised five identical specimens to ensure the reliability of the results. In this study, the specimens were divided into four groups; the first group, represented by the NC series, was unconfined and served as the reference group to evaluate the effectiveness of confinement. The second group included the FC1, PC2.0, and PC0.2 series, each reinforced with an equivalent of one layer of CFRP. The FC1 series was fully confined using one continuous wrap, whereas PC2.0 and PC0.2 received partial confinement applied in two separate strips. The key difference lies in the starting position of these partial layers: PC2.0 had wraps starting from the confined ends, while PC0.2 had them starting from the unconfined ends. The main objective, with or without end confinement, is to investigate whether stress concentration develops at the ends of the confined concrete cylinders. If no such concentration is observed, the spacing between the CFRP strips is progressively reduced to evaluate its influence on confinement effectiveness.

**Figure 1:**
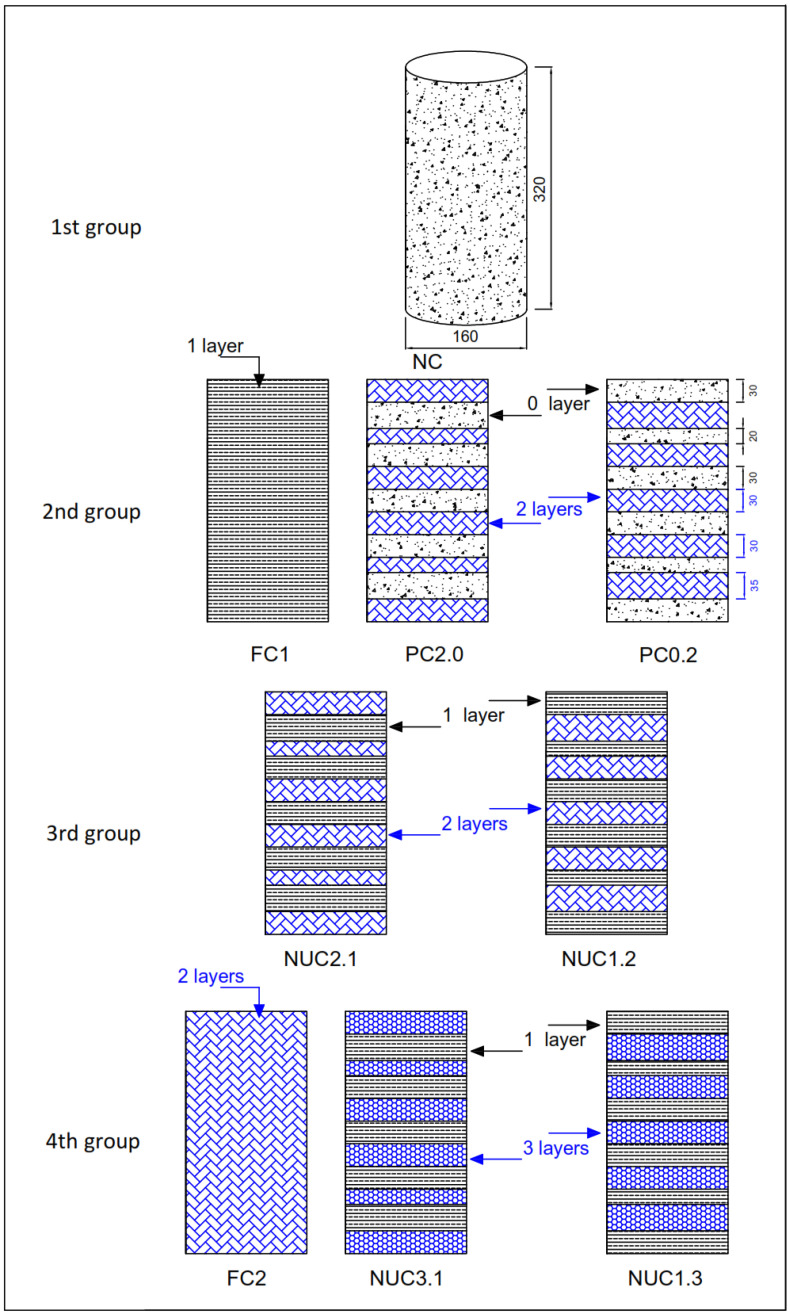
Details of the studied group configurations (unit: mm).

The third group, composed of NUC2.1 and NUC1.2, featured non-uniform confinement using 1.5 equivalent layers, achieved by combining a full layer with a partial one. In NUC2.1, the partial wrap began at the confined ends, whereas in NUC1.2, it started at the unconfined ends. Lastly, the fourth group included FC2, NUC3.1, and NUC1.3, all reinforced with an amount of CFRP equivalent to two full layers. FC2 was entirely wrapped using two continuous layers, and the NUC3.1 and NUC1.3 series combined a full layer with two partial layers, initiated from the confined ends in NUC3.1, and from the unconfined ends in NUC1.3. The series naming convention comprises two parts: the first part includes the letters NC, FC, PC, and NUC; the second part indicates the number of CFRP layers for partially wrapped and non-uniformly confined specimens. For instance, in NUC1.3, the bonding strips have three layers, while the covering strips have one (Figure 1, Table 3).

### 2.3. Test Setup and Instrumentation

**Figure 2:**
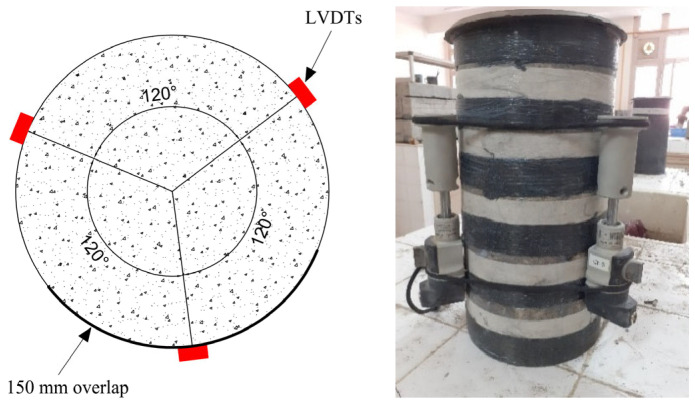
Linear variable displacement transducers.

Compression tests were conducted using an MCC8 hydraulic press with a 3000 kN capacity. The specimens were subjected to three preloading cycles, with stress levels ranging from 0.5 MPa to one-third of the previously determined compressive strength of the concrete. Both loading and unloading were conducted at a constant rate of 0.5 MPa/s. These preliminary cycles were performed to verify the proper alignment of the specimens within the testing apparatus and to determine the initial elastic modulus, followed by a final test with monotonic axial compression at a constant load rate of 0.5 MPa/s until failure. Before applying CFRP, all specimens were cleaned with compressed air. The tests were conducted under controlled laboratory conditions with an ambient temperature of 23 ± 2 °C and relative humidity of 55 ± 5%. Markings were made with tape for the PC series and on the first CFRP layer for the NUC series to guide the strip layout. The adhesive used was a two-component epoxy system (resin and hardener in a 4:1 ratio, as specified by the supplier), applied to both the concrete surface and the CFRP fabric. CFRP strips were oriented circumferentially with a 150 mm overlap at joints to prevent premature debonding. All specimens were cured for 72 h prior to testing. For series PC2.0 and PC0.2, the strip position was marked on the concrete surface using tape. For series NUC2.1, NUC1.2, NUC3.1, and NUC1.3, markings were made on the first CFRP layer (Figure 2).

After wrapping, specimens were capped with a sulfur compound to ensure uniform load distribution. For testing, three linear variable displacement transducers (LVDTs) (1 µm resolution, 150 mm gauge length) were mounted 120° apart around each specimen and connected to a computer-based data acquisition system. Uniaxial compression tests were performed on cylindrical specimens following the NF P 18-406 standard [46]. The compressive strength of the concrete and the modulus of elasticity were determined by averaging the results obtained from identical specimens.

### 2.4. Descriptive Statistical Measures

A comprehensive statistical analysis was performed to evaluate the accuracy and robustness of the proposed predictive models for confined concrete behavior. Several performance metrics were employed to assess the predictive capability of the models for confined compressive strength and ultimate axial strain:

R-squared (R^2^) quantifies the proportion of variance in the experimental data explained by the model. A value closer to 1.0 indicates a better fit, as shown in Equation (1).

(1) $$R^{2}=1-\frac{\sum_{i=1}^{n}{\left(y_{i}-{\hat{y}}_{i}\right)}^{2}}{\sum_{i=1}^{n}{\left(y_{i}-\bar{y}\right)}^{2}}$$

The adjusted R-squared (${R_{adj}}^{2}$) accounts for the number of predictors in the model and adjusts R^2^ accordingly, preventing overfitting, indicated in Equation (2).

(2) $${R_{adj}}^{2}=1-\frac{\left(1-R^{2}\right)\left(n-1\right)}{n-P-1}$$

Root Mean Squared Error (RMSE) measures the average magnitude of prediction errors, with larger errors penalized more heavily. Lower RMSE values indicate better model performance, as shown in Equation (3).

(3) $$RMSE=\sqrt{\frac{1}{n}\sum_{i=1}^{n}{\left(y_{i}-{\hat{y}}_{i}\right)}^{2}}$$

Mean Absolute Error (MAE) provides the average of the absolute differences between predicted and observed values. While it is less sensitive to outliers than RMSE, it still reflects the overall accuracy, as shown in Equation (4).

(4) $$MAE=\frac{1}{n}\sum_{i=1}^{n}\left|y_{i}-{\hat{y}}_{i}\right|$$

Scatter Index (SI) is a normalized metric obtained by dividing the RMSE by the mean of the observed values. Lower SI values denote higher predictive accuracy relative to the data range, as shown in Equation (5).

(5) $$SI=\frac{RMSE}{\bar{y}}$$

The a20-index represents the proportion of predictions where the relative error is within ±20% of the experimental value. This metric helps assess practical reliability by highlighting how frequent predictions fall within acceptable error bounds, indicated in Equation (6).

(6) $$a20-index=\frac{m20}{M}$$

where n is the number of observations, $y_{i}$ is the observed value, ${\hat{y}}_{i}$ is the predicted value, $\bar{y}$ is the mean of observed values, P is the number of model parameters, M is the total number of samples, and m20 is the number of samples with relative error ≤ 20%.

## 3. Results and Discussion

### 3.1. Failure Modes

**Figure 3:**
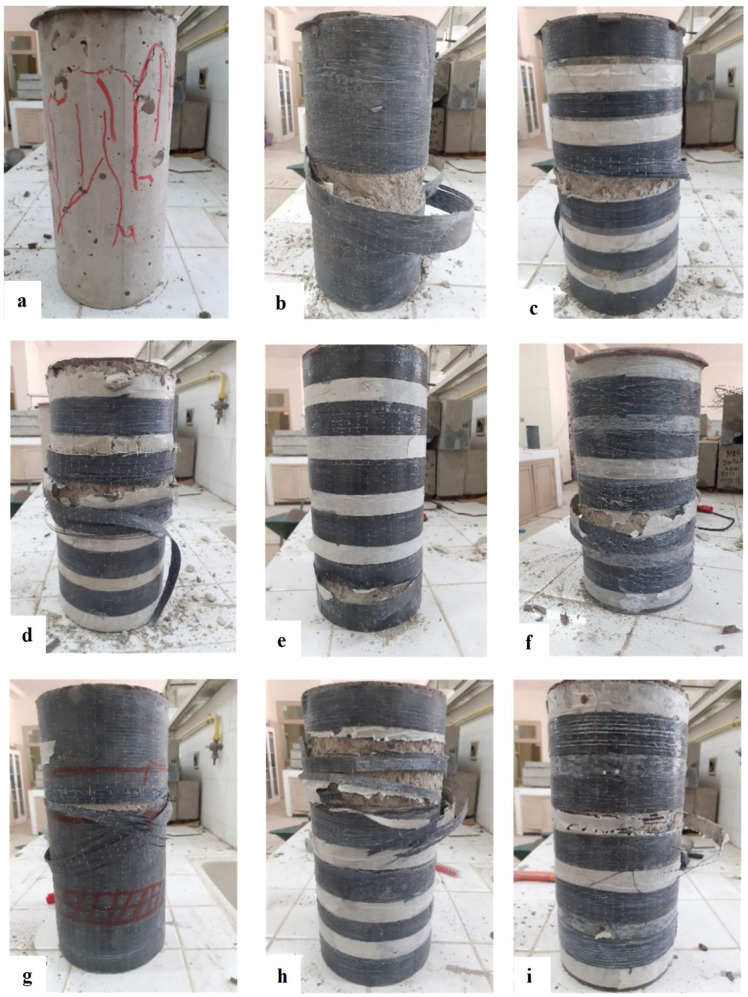
Failure modes of various CFRP-wrapped concrete series. (**a**) NC cylinders, (**b**) FC1 cylinders, (**c**) PC0.2 cylinders, (**d**) PC2.0 cylinders, (**e**) NUC2.1 cylinders, (**f**) NUC1.2 cylinders, (**g**) FC2 cylinders, (**h**) NUC3.1 cylinders and (**i**) NUC1.3 cylinders.

**Figure 4:**
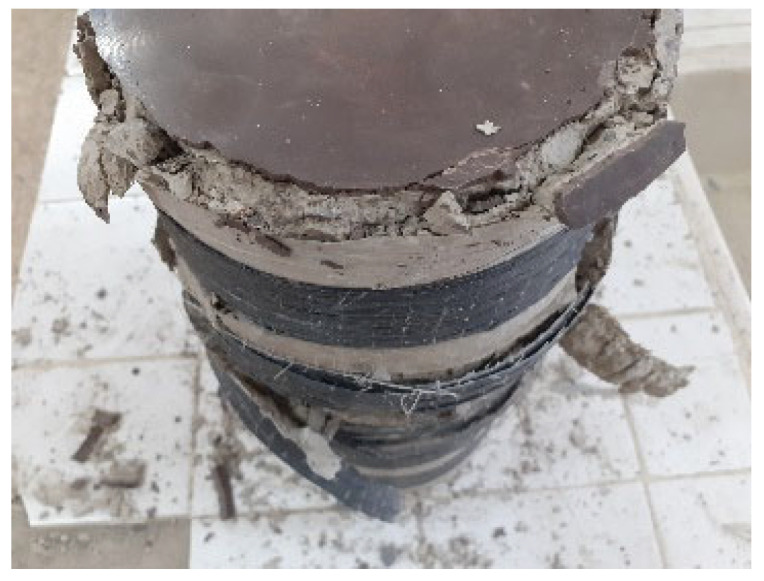
Concrete cracking at upper- and lower-cylinder ends of series PC0.2.

The failure mechanisms observed across the tested specimens varied by confinement type:

- NC—Brittle Failure Mode: The unconfined concrete cylinders (NC) exhibited a brittle failure behavior, which is typical of plain concrete without lateral restraint. Initial cracks appeared randomly in various regions of the specimen, with some emerging on the surface and others initiating deep within the concrete core. As the applied load increased, these cracks extended and eventually interconnected, forming a critical fracture network. This led to a sudden crushing and disintegration of the concrete, accompanied by localized spalling and abrupt collapse. Such behavior indicates that the material had very limited post-peak deformation capacity, failing shortly after reaching its maximum compressive strength. This failure mode is characteristic of quasi-brittle materials with no or minimal confinement, and is consistent with observations reported by Abdellah et al. [45] and Michael et al. [47] (see Figure 3a).
- The fully confined (FC) concrete cylinders exhibited a brittle and catastrophic failure mode, predominantly characterized by the sudden rupture of the CFRP jacket, typically occurring near mid-height. This localized rupture signifies a critical loss of confinement pressure, attributable to the lateral expansion of the concrete core exceeding the tensile capacity of the composite wrap at that specific location. The abrupt nature of the failure, without prior significant warning, reflects the limited energy absorption and ductility of the CFRP system under high confinement-induced hoop stresses. The failure zone at mid-height may also be associated with strain localization, non-uniform axial stress distribution, which collectively promote stress concentrations within the jacket. This behavior aligns with observations reported in the literature [36], where CFRP-confined specimens exhibited premature jacket rupture due to inadequate strain compatibility between the concrete core and the external reinforcement. The anisotropic and brittle nature of CFRP materials further limits their capacity to undergo plastic deformation, resulting in a rupture that is predominantly elastic brittle in character. The observed failure morphology, as depicted in Figure 3b,g, underscores the critical role of composite tensile properties and confinement efficiency in determining the ultimate axial performance of confined concrete. These findings highlight the necessity of incorporating local strain behavior, fiber orientation, and composite fracture mechanics into the design and modeling of fully confined systems to improve structural resilience and predictability.
- The partially confined (PC) concrete cylinders exhibited a progressive failure mechanism initiated by the rupture of one or more discrete CFRP strips, as illustrated in Figure 3c,d. The fracture sequence began with localized overstressing of the jacketed regions, followed by partial loss of lateral confinement, which accelerated axial deformation and concrete crushing in unconfined zones. Notably, a distinct behavioral divergence was observed between configurations PC2.0 and PC0.2, emphasizing the critical influence of confinement placement. In the PC2.0 specimens, where the confinement was concentrated near the ends of the cylinder, failure occurred without observable crushing at the extremities, indicating that end zone confinement played a pivotal role in delaying or mitigating damage propagation. Conversely, in PC0.2, where the ends were insufficiently confined, premature crushing and spalling were clearly evident in those regions (Figure 4). This contrast underscores the importance of strategic confinement distribution and validates earlier findings by Pham et al. [35], which emphasized the sensitivity of concrete behavior to localized confinement effects. The results demonstrate that even partial confinement, when properly positioned, can significantly enhance axial performance by suppressing early dilation and crack coalescence, particularly in regions prone to stress concentration and instability.
- In the case of non-uniformly confined (NUC) specimens, failure was typically initiated in geometrically or structurally weak regions, most frequently observed at locations where a single layer covering strip was applied or at the interface between overlapping and bonded CFRP strips, as depicted in Figure 3e–i. These zones consistently exhibited the earliest signs of distress, highlighting a critical vulnerability associated with interfacial bonding deficiencies between adjacent CFRP layers.
- The covering strips, which are applied externally without direct bonding pressure, tend to exhibit inferior adhesion and reduced confinement efficiency, making them the most fracture-prone components of the system. The presence of these discontinuities introduces strain incompatibility and stress concentration zones, which facilitate early crack initiation and propagation under axial loading. These findings are in strong agreement with the observations reported by Junlong et al. [37], who also identified bond quality and strip discontinuity as key factors limiting the structural effectiveness of non-uniform confinement schemes. The experimental results underscore the necessity of improving composite bonding strategies and optimizing strip layout configurations to ensure continuity in confinement and delay the onset of localized failures.

### 3.2. Stress–Strain Curves

**Figure 5:**
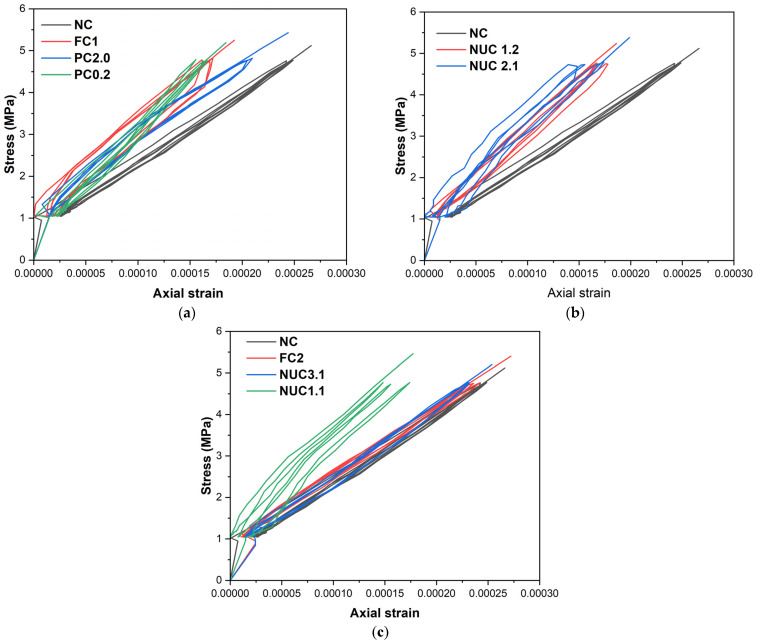
Stress–strain curves for the series under cyclic loading. (**a**) Full confinement, (**b**) Partial confinement and (**c**) Non-uniform confinement.

All specimens exhibited a consistent linear stress–strain response during initial cyclic loading up to 30% of peak load (Figure 5). The results indicate that strain increases proportionally with the load and decreases as the load is reduced, with all four load–unload cycles exhibiting the same slope. This phase primarily reflected the elastic behavior of the concrete; CFRP influence was negligible due to limited lateral expansion. This lack of impact is likely attributed to the resin’s behavior, as fibers do not contribute to the observed elasticity.

**Figure 6:**
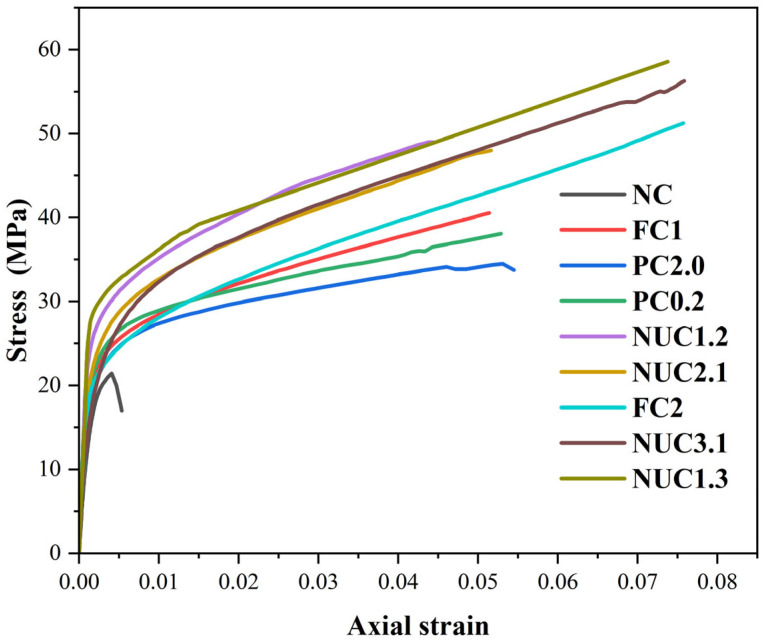
Stress–strain curves for the series under monotonic loading.

Under monotonic compression (Figure 6), unconfined specimens showed typical brittle behavior: linear elasticity followed by sudden strength loss. In contrast, CFRP-confined cylinders exhibited significant enhancements in strength and ductility. The curves for the reinforced series can be divided into two phases as [48,49]. The pre-peak linear phase is controlled by concrete stiffness, and the post-yield confinement phase reveals that lateral dilation activates CFRP confinement, resulting in a ductile response with no distinct peak [43]. The lateral deformations of the concrete under axial load induce outward expansion, which is restrained by the CFRP confinement. This restraint generates lateral confining pressure from the CFRP jacket, which in turn delays the initiation of crushing, enhances energy absorption, increases the axial compressive strength, and produces a pronounced plastic plateau in the stress–strain response. These mechanisms are consistent with the findings of [50] and other relevant studies.

### 3.3. Axial Strength and Deformation Enhancement

**Figure 7:**
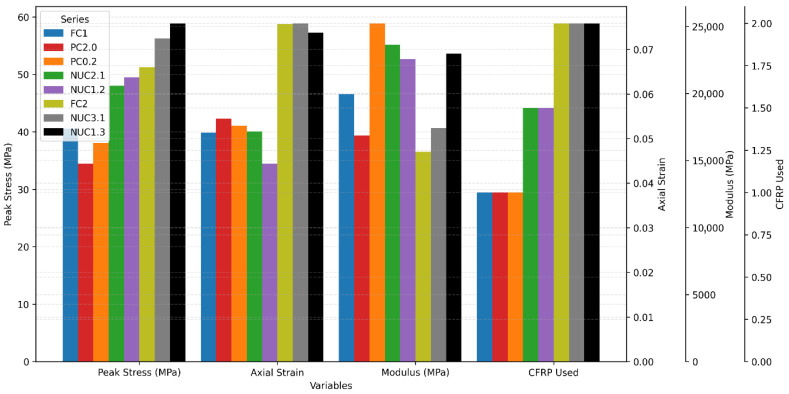
Performance evaluation of confined series: strength, strain, elasticity, and CFRP consumption.

Table 4 and Figure 7 summarizes the strength and strain improvements for all wrapped series compared to the NC reference. The calculated coefficients of variation (COV) for the measured peak stresses ranged from 0.22% to 0.48%, indicating a very low dispersion of individual values around the mean. This limited variability suggests a high degree of material homogeneity and highlights the consistency of experimental procedures. In particular, the series confined with composite fabrics (NUC3.1 and NUC1.3) exhibited the lowest COV values, further confirming the repeatability of the test protocol and the overall reliability of the experimental campaign.

However, the coefficients of variation (COVs) for peak axial strain range from 1.66% to 5.17%, depending on the confinement configuration. Once again, the NUC3.1 and NUC1.3 series show the lowest COVs—1.66% and 2.10%, respectively—indicating excellent consistency in peak strain values, which can be attributed to the stabilizing effect of the external CFRP confinement. In contrast, the PC2.0 (5.17%) and FC2 (4.20%) series exhibit greater variability in axial strain, possibly due to localized stress concentrations or early debonding phenomena that introduce strain heterogeneity.

**Figure 8:**
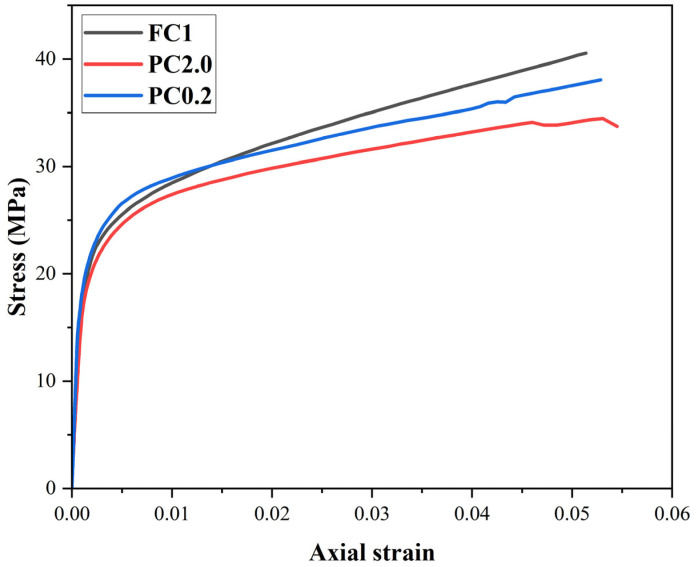
Stress–strain curves of FC1, PC2.0, and PC0.2.

The CFRP composite wrapping significantly enhanced the maximum strength. FC1 (fully confined, 1 layer) improved strength by 89% and deformation by 1159%. PC2.0 and PC0.2 (partial confinement) achieved 61% and 78% strength gains, respectively. The maximum strength values for series PC2.0 and PC0.2 are −15% and −6% than those of FC1, respectively (Figure 8 and Table 5). However, the recorded deformations for PC2.0 and PC0.2 exceed those for FC1 by 6% and 3%, respectively. These trends confirm that full wrapping provides higher compressive strength, and the partial confinement enhances deformability [45,51].

Additionally, a comparison between the FC2 and FC1 series revealed increases in ultimate compressive strength and ultimate axial deformation by 26% and 47%, respectively, highlighting the effect of the amount of CFRP material on the behavior of concrete cylinders within the same confinement type. Similar trends are observed when comparing the series (PC2.0, NUC2.1, and NUC3.1) with (PC0.2, NUC1.2, and NUC1.3). These findings indicate that increased lateral pressure from the CFRP composite correlates with the amount of material used, leading to enhanced performance of the confined cylinders [52].

**Figure 9:**
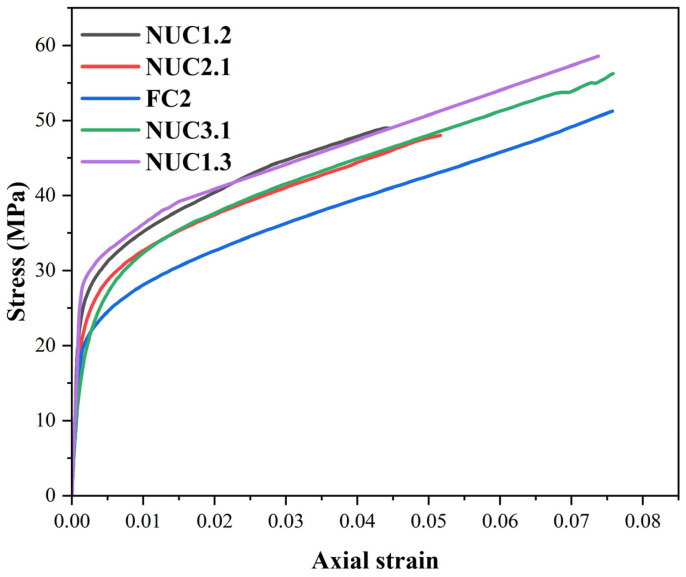
Stress–strain curve for series NUC1.2, NUC2.1, FC2, NUC3.1, and NUC1.3.

Series FC2 (two layers full confinement) is compared with NUC3.1 and NUC1.3, which use the same CFRP amount but in a non-uniform layout (Figure 9 and Table 6).

The results reveal that NUC3.1 is 10% higher in strength and has comparable deformation (0%), NUC1.3 is 15% higher in strength and has slightly reduced deformation (–3%). This confirms that optimized non-uniform layouts outperform full confinement in strength without compromising ductility [36,37].

Figure 9 and Table 6 also show the behavior of the NUC2.1 and NUC1.2 series compared to the FC2 series. NUC2.1 and NUC1.2 series use 1.5 times the amount of CFRP required for full confinement, while the FC2 series uses twice the amount. Despite minor strength reductions (6% and 3%, respectively), deformation dropped significantly (32% and 41%). This demonstrates the superior material efficiency of non-uniform confinement. NUC1.2 and NUC1.3 showed better strength but lower ductility than their counterparts with thicker outer strips (NUC2.1 and NUC3.1), reinforcing the importance of confinement strip positioning and overlap density [38,53].

### 3.4. Proposed Model

When concrete is confined using composite wraps like FRP (fiber-reinforced polymer), its compressive strength increases due to the lateral pressure generated by the wrap’s resistance to the concrete’s transverse expansion. Traditionally, most theoretical models such as those in the design code (FIB Bulletin) [42] or by Wang et al. [34] describe the confined concrete’s compressive strength as a linear or semi-linear function of the unconfined concrete strength and the effective confinement [17,37]. The key parameter in these models is the FRP-generated confinement pressure, which primarily depends on the following:

- The FRP’s tensile strength;
- The lateral reinforcement ratio (number and distribution of FRP layers);
- The FRP’s elastic modulus;
- The number of plies used.

A new confined model was developed to predict the confined compressive strength ($f_{cc}$) and the ultimate strain ($\varepsilon_{cu}$) in CFRP-confined concrete cylinders made from low-strength concrete. This model considers the combined effects of material properties and confinement configuration, including full, partial, and non-uniform CFRP layouts under equal material usage; see Equations (7) and (8):

(7) $$f_{cc}=f_{co}+K_{1}\times f_{fRFC}\times \rho_{f}\;$$

(8) $$\varepsilon_{cu}=\varepsilon_{co}+K_{2}\times \varepsilon_{fu}\times \rho_{f}$$

where $f_{co}$ is the unconfined compressive strength of concrete, $f_{fRFC}$ is the tensile strength of CFRP, $\varepsilon_{co}$ is the ultimate strain of unconfined concrete, $\varepsilon_{fu}$ is the ultimate strain of CFRP, and $\rho_{f}$ is the volumetric ratio of CFRP; see Equation (9). The coefficients K_1_ and K_2_ are empirical parameters that were calibrated in this study using a regression analysis based on the experimental results obtained from the full, partial, and non-uniform confinement configurations. Their final values, shown in Table 7, reflect the influence of confinement distribution on strength and ductility enhancement.

(9) $$\rho_{f}=\frac{\sum \left(b_{fi}\times .n_{i}\right)}{\left(\sum S_{i}\right)\beta +\sum \left(b_{fi}\right)}$$

where $b_{fi}$ is the CFRP strip width, $n_{i}$ is the number of CFRP layers, and $S_{i}$ is the spacing between strips. The coefficient β accounts for the effect of the confinement distribution along the cylinder height Equation (10):

(10) $$\beta =\frac{\sum S_{i}}{\sum \left(b_{fi}\right)}$$

The model proposed in this study is a simplified, yet physically grounded development based on confinement mechanics:

- A linear relationship was adopted because confined concrete’s strength typically increases linearly with confinement pressure in most experimental observations.
- Adjustable coefficients K_1_ and K_2_ were introduced to account for the effect of FRP layer distribution and arrangement, an aspect often overlooked by classical models that assume uniform, continuous confinement.
- The following offers a comparison:
- The FIB Bulletin [42] defines confinement pressure as a function of the wrap’s elastic modulus and thickness, assuming uniform distribution.
- Wang et al. [34] model uses a semi-linear relationship between compressive strength, ultimate strain, confinement pressure, and unconfined concrete strength.

The model’s validity was assessed by comparing its theoretical predictions with experimental results for both compressive strength and axial strain. This evaluation also included data from Saci et al. [51], who examined concrete cylinders wrapped with CFRP sheets placed at different spacings (30 mm, 45 mm, and 65 mm), identified as CPH30, CPH45, and CPH65, respectively. Table 8 presents the predicted and experimental values, together with the corresponding relative errors, to illustrate the model’s accuracy across varying confinement configurations. Table 9 summarizes the results for both compressive strength and strain prediction.

These values presented in Table 9 confirm the model’s high predictive accuracy and robustness, particularly in estimating confined strength. Strain predictions also showed good accuracy despite greater inherent variability in strain data.

**Figure 10:**
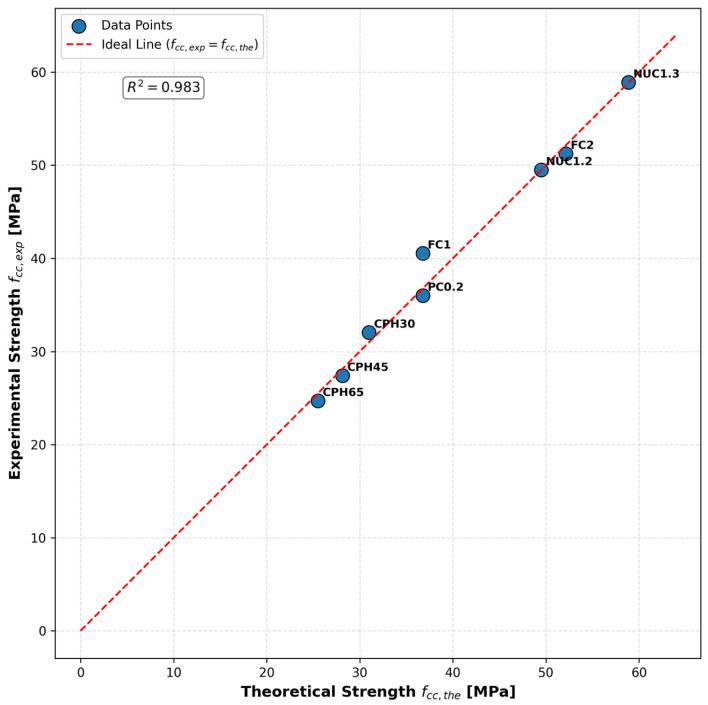
Correlation between experimental and predicted peak compressive stress.

The comparison between the experimental and theoretical peak compressive stress values $f_{cc}$ is illustrated in Figure 10. The data points closely follow the ideal line $f_{cc,exp}=f_{cc,the}$, indicating a strong agreement between the proposed model predictions and the observed experimental results. This observation is further supported by a high coefficient of determination, $R^{2}=0.983$, which confirms the predictive accuracy of the model across different confinement configurations.

**Figure 11:**
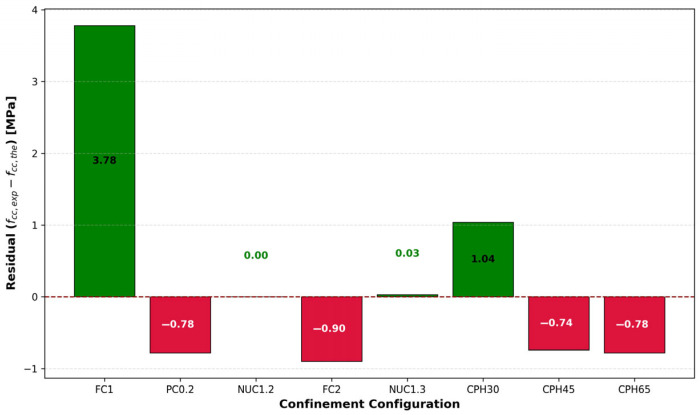
Residual distribution of predicted vs. experimental compressive strength for various confinement configurations.

Despite the strong correlation, minor deviations can be observed in specific configurations, such as FC1 and CPH65. To better illustrate these deviations, the residual plot in Figure 11 presents the differences between experimental and predicted values ${(f}_{cc,exp}-f_{cc,the})$ for each configuration. The residuals remain within a narrow range (±4 MPa), suggesting that the model does not systematically overestimate or underestimate the strength values. In fact, the largest absolute deviation does not exceed approximately 3.8 MPa, which is within an acceptable margin for practical engineering applications.

Furthermore, the residuals are symmetrically distributed around zero, with no discernible trend or bias relative to the type of confinement. This reinforces the robustness and generalizability of the proposed model across full, partial, and non-uniform confinement scenarios.

Overall, the close match between experimental and theoretical values, combined with the small and randomly distributed residuals, validates the effectiveness of the proposed model for estimating the confined concrete strength under varying confinement conditions.

Table 10 presents the ANOVA results to validate the significance of the predictive models. With *p*-values well below 0.05, both models are statistically significant and not a result of random variation. The proposed confinement model offers high accuracy in predicting the mechanical behavior of CFRP-confined concrete cylinders across various wrapping configurations. Its strength lies in incorporating confinement geometry and material distribution, making it a practical and adaptable tool for structural design and optimization. To further validate the proposed confinement model, its predictions were compared with those from the FIB Model Code [42] and the model by Wang et al. [34].

### 3.5. Analytical Verification

In this study, the compressive strength and ultimate deformation of series FC1, PC0.2, NUC1.2, FC2, and NUC1.3 along with series CPH30, CPH45, and CPH65 from Saci et al. [51] and Abdellah et al. [45] are calculated using the models proposed by Wang et al. [34] and the design code (FIB Bulletin) [42]. Equations have been developed for total and partial confinement scenarios (Table 11). For calculations based on [39], the effectiveness factor for CFRP composite strain ($k_{\varepsilon }$) has been assumed equal to 0.75 according to [54]. Conversely, Wang et al. [34] propose $k_{\varepsilon }=0.586$ for the CFRP composite strain. The effective strain of the bonded CFRP sheet at failure ($\varepsilon_{fe}$) is presented in Equation (11) [42].

(11) $$\varepsilon_{fe}=k_{\varepsilon }\times \varepsilon_{fu},$$

For non-uniform confinement (NUC1.2 and NUC1.3), confinement pressure was computed as the superposition of the effects from full and partial wrapping layers.

Table 12 presents predicted versus experimental values for compressive strength and strain using all models, along with relative errors.

The proposed model consistently delivered predictions with lower relative errors and strong agreement with experimental values, particularly for non-uniform configurations where the other models showed reduced accuracy. Table 13 shows a summary of statistical indicators.

A detailed comparison was performed between the predictive of proposed model, the FIB bulletin [42], and Wang et al. [34], focusing on their ability to estimate the peak confined compressive stress and the corresponding axial strain in concrete. As presented in Table 13, the developed model displayed superior performance across all evaluated criteria. The correlation with experimental values was particularly strong, with coefficients of determination reaching 0.983 for stress and 0.905 for strain, while the adjusted R^2^ values (0.971 and 0.867) indicate the model’s capacity to generalize across different test conditions.

In terms of error analysis, the proposed model achieved the lowest discrepancies between predicted and observed values. The RMSE was calculated as 1.497 MPa for stress and 0.0067 for strain, while the MAE reached 1.006 MPa and 0.006, respectively. The SI values, which help evaluate the relative dispersion of predicted values, were also very low (0.037 for stress and 0.0012 for strain), underlining the consistency and reliability of the model.

In contrast, the FIB model delivered moderate accuracy, particularly for stress prediction (R^2^ = 0.817; SI = 0.077), but it was less effective for strain (R^2^ = 0.576; SI = 0.141), indicating its limited capacity to capture nonlinear deformation mechanisms. The poorest performance was recorded for the Wang et al. model, especially regarding strain, with a very low R^2^ of 0.152 and a high SI of 0.301, indicating poor predictive alignment with experimental outcomes.

The proposed model outperformed the FIB Bulletin and the models by Wang et al. in all statistical measures, particularly in strain prediction, where other models showed significant deviations and low explanatory power. The statistical analysis demonstrates a strong agreement between the proposed model and experimental observations while highlighting its clear advantage over existing design approaches. By effectively incorporating the influence of confinement geometry and material utilization, the model serves as a valuable and practical asset for structural engineering tasks, especially when tackling the complexity of optimizing non-uniform CFRP confinement layouts.

Although the proposed model demonstrated superior performance compared to existing models, particularly in predicting longitudinal strain, these results should be interpreted with caution. The experimental dataset used in this study is limited in both size and diversity, which may affect the generalizability of the findings. This constraint may partly explain the observed discrepancies with existing models, which were not originally calibrated for non-uniform confinement scenarios. In contrast, the proposed model was specifically tailored to the three confinement configurations studied herein (full, partial, and non-uniform), which likely contributed to its improved accuracy within the defined experimental context. To enhance the model’s reliability and extend its applicability to real structural elements, further research is recommended. This includes expanding the experimental database by increasing the number of tested specimens and incorporating a broader range of confinement geometries, material properties, and loading conditions. Additionally, multi-scale testing, ranging from material-level specimens to structural components, would help clarify the relationship between local confinement behavior and overall structural performance. Finally, full-scale concrete column tests under realistic boundary conditions, including eccentric or combined loading, are essential to validate the model’s predictive capacity in practical applications. These efforts will strengthen the reliability of confinement models and support their integration into modern structural design and retrofitting guidelines.

## 4. Conclusions

CFRP composites for concrete confinement have gained widespread attention due to their high strength-to-weight ratio and corrosion resistance. However, there remains a critical gap in the literature: a lack of systematic, controlled comparisons between full, partial, and non-uniform confinement schemes under identical material and boundary conditions. This study addresses that gap through a combined experimental and analytical investigation of the axial behavior of low-strength concrete cylinders confined with different CFRP configurations. All configurations were applied using equal or proportionally scaled amounts of CFRP, enabling a direct and quantitative comparison of structural efficiency per unit of material used. The key findings of this work are as follows:

- All CFRP-confinement schemes significantly enhanced compressive strength and axial deformation, with the most pronounced gains observed in non-uniform configurations, which outperformed the fully wrapped specimens.
- Non-uniformly confined cylinders achieved up to 14.93% higher compressive strength than fully confined ones using the same CFRP quantity. Even with a 25% reduction in CFRP, performance loss remained minimal, demonstrating a favorable balance between performance and material economy.
- While partial wrapping provided lower strength than full confinement, it provides comparable or superior ductility, suggesting that it is appropriate where deformation capacity is critical.
- A new confinement model was developed and statistically validated, outperforming existing formulations (e.g., FIB Bulletin, Wang et al.) in predicting both strength and strain. The model achieved R^2^ values of 0.983 (strength) and 0.905 (strain), with low RMSE and scatter index values. ANOVA analysis confirmed the model’s statistical robustness.
- The model introduces two calibration parameters, K_1_ and K_2_, which allow adaptation to different confinement types, including non-uniform layouts rarely addressed by classical models. This extends its relevance to real-world strengthening applications where full wrapping is impractical. In contrast to prior studies that evaluated single confinement types in isolation, this research delivers a unified experimental platform and a generalized analytical model for comparing CFRP schemes under controlled conditions.

These findings have direct practical implications for the rehabilitation of aging and deficient concrete structures, especially in scenarios involving low-strength concrete and limited material resources. By demonstrating that strategically placed, non-uniform CFRP confinement can outperform conventional full wrapping, this study introduces a new paradigm for resource-efficient, performance-oriented confinement design.

Although the model developed in this study demonstrated high predictive accuracy for confined strength and strain, its applicability is currently limited to small-scale cylindrical specimens under idealized conditions. As such, its direct use in structural-scale prediction should be approached with caution. To bridge this gap, future research should focus on validating the model using full-scale structural elements, incorporating realistic boundary conditions, loading eccentricities, and long-term environmental effects. Integrating the model’s confinement coefficients into finite element simulations and developing correction factors for field applications will be essential steps toward reliable structural-level implementation.

## Figures and Tables

**Table 1:** Concrete mix characteristics.

Component	Value
Cement (kg/m^3^)	210
Water (L/m^3^)	160
Gravel 3/8 (kg/m^3^)	218.68
Gravel 8/15 (kg/m^3^)	977.06
Crushed Sand 0/3 (kg/m^3^)	847.24
Water/Cement ratio	0.761
$\mathrm{Compressive}\;\mathrm{strength},\;f_{co}$(MPa)	21.42
$\mathrm{Ultimate}\;\mathrm{axial}\;\mathrm{strain},\;\varepsilon_{co}(\%)$	0.408

**Table 2:** Mechanical properties of Sika Wrap^®^-230C/45.

Properties	Value
Tensile strength (GPa)	4.3
Tensile E-modulus (GPa)	234
Elongation at break	1.8%
Fiber density (g/cm^3^)	1.76
Fabric design thickness (mm)	0.13

**Table 3:** Details of the studied configurations.

Group	Series	Equivalent Layers	Confinement Type	Description	
1	NC	0	unconfined	/	/
2	FC1	1	full	1 full layer	/
	PC2.0	1	partial	/	2 partial layers (start: confined ends)
	PC0.2	1	partial	/	2 partial layers (start: unconfined ends)
3	NUC2.1	1.5	non-uniform	1 full layer	1 partial layer (start: confined ends)
	NUC1.2	1.5	non-uniform	1 full layer	1 partial layer (start: unconfined ends)
4	FC2	2	full	2 full layers	/
	NUC3.1	2	non-uniform	1 full layer	2 partial layers (start: confined ends)
	NUC1.3	2	non-uniform	1 full layer	2 partial layers (start: unconfined ends)

**Table 4:** Results of wrapped concrete cylinders from different series compared to NC.

Series	$f_{cc,exp}$ (MPa)	COV (%)	Relative Strength Gain (%)	$\varepsilon_{cu,exp}$	COV (%)	Relative Strain Gain (%)	E (MPa)
FC1	40.56	0.47	89%	0.05138	3.20	1159%	19,932
PC2.0	34.48	0.32	61%	0.05450	5.17	1236%	16,866
PC0.2	38.07	0.29	78%	0.05286	2.56	1196%	25,231
NUC2.1	48.01	0.42	124%	0.05161	2.79	1165%	23,634
NUC1.2	49.50	0.48	131%	0.04438	2.59	988%	22,559
FC2	51.24	0.37	139%	0.07572	4.20	1756%	15,650
NUC3.1	56.27	0.41	163%	0.07584	1.66	1759%	17,428
NUC1.3	58.89	0.22	175%	0.07376	2.10	1708%	22,968

**Table 5:** Test results of series FC1, PC2.0, and PC0.2 compared to FC1.

Series	Q (kN)	$f_{cc,exp}$ (MPa)	Percentage Difference	$\varepsilon_{cu,exp}$	Percentage Difference
FC1	815.09	40.56	/	0.05138	/
PC2.0	692.91	34.48	−15%	0.05450	6%
PC0.2	765.05	38.07	−6%	0.05286	3%

**Table 6:** Test results of NUC1.2, NUC2.1, NUC3.1, and NUC1.3 compared to FC2.

Series	Q (kN)	$f_{cc,exp}$ (MPa)	Percentage Difference	$\varepsilon_{cu,exp}$	Percentage Difference
FC2	1029.72	51.24	/	0.07572	/
NUC2.1	964.81	48.01	−6%	0.05161	−32%
NUC1.2	994.75	49.50	−3%	0.04438	−41%
NUC3.1	1130.80	56.27	10%	0.07584	0%
NUC1.3	1183.45	58.89	15%	0.07376	−3%

**Table 7:** Influence of confinement regime on the constitutive coefficients K_1_ and K_2_ in cylindrical elements subjected to axial compression.

Type of Confinement	$K_{1}$	$K_{2}$
Full	0.032	2.04
Partial	0.032	2.49
Non-uniform	0.039	1.54

**Table 8:** Prediction results using the proposed model compared to experimental results.

	Type of Confinement	Configuration	$f_{cc,exp}$ (MPa)	$f_{cc,the}$ (MPa)	Relative Error	$\varepsilon_{cu,exp}$	$\varepsilon_{cu,the}$	Relative Error
Results obtained in this study	**Full**	FC1	40.56	36.78	−0.0932	0.0514	0.04488	−0.1268
	**Partial**	PC0.2	36.00	36.78	0.0217	0.0529	0.05388	0.0185
	**Non-uniform**	NUC1.2	49.50	49.50	0.0000	0.0444	0.05028	0.1324
	**Full**	FC2	51.24	52.14	0.0176	0.0757	0.08568	0.1318
	**Non-uniform**	NUC1.3	58.89	58.86	−0.0005	0.0738	0.06568	−0.1100
Results Obtained by Dahmani et al. [51]	**Partial**	CPH30	32.03	30.99	−0.0325	0.0290	0.03511	0.2105
	**Partial**	CPH45	27.40	28.14	0.0272	0.0268	0.02588	−0.0342
	**Partial**	CPH65	24.71	25.49	0.0314	0.0084	0.01726	1.0548

**Table 9:** Statistical summary of model performance.

Measure	Confined Stress	Confined Strain
R^2^	0.983	0.905
Adjusted R^2^	0.971	0.867
RMSE	1.497	0.0067
Mean response	40.33	0.0453
Observations (or weighted sums)	8	8

**Table 10:** ANOVA analysis for the studied responses.

Experimental Layout	Source	Degree of Freedom	Sum of Squares	Mean Square	F Rapport
Confined stress	Model	1	1026.717	1026.717	343.268
	Error	6	17.946	2.991	Prob. > F
	Uncorrected Total	7	1044.663		0.00001
Confined strain	Model	1	0.00344	0.00344	57.333
	Error	6	0.00036	0.00006	Prob. > F
	Uncorrected Total	7	0.0038		0.002

**Table 11:** Summary of design code and model for CFRP-confined concrete.

Model	Strength	Axial Deformation
FIB Bulletin [42]	$f_{cc}=f_{co}\left(0.2+3\sqrt{\frac{f_{l}}{f_{co}}}\right)$	$\varepsilon_{cu}=\varepsilon_{co}\left(2+1.25\frac{E_{c}}{f_{co}}\varepsilon_{fe}\sqrt{\frac{f_{l}}{f_{co}}}\right)$
Wang et al. [34]	$f_{cc}=f_{co}\left(1+3.3k_{e}\frac{f_{l}}{f_{co}}\right)$	$\varepsilon_{cu}=\varepsilon_{co}\left(1.75+12\left(\frac{f_{l}}{f_{co}}\right){\left(\frac{\varepsilon_{h,rup}}{\varepsilon_{co}}\right)}^{0.45}\right)$

**Table 12:** Prediction accuracy comparison.

Configuration	Experimental		FIB Bulletin [42]				Wang et al. [34]			
	$f_{cc,exp}$(MPa)	$\varepsilon_{cu,exp}$	$f_{cc,the}$(MPa)	Relative Error	$\varepsilon_{cu,the}$	Relative Error	$f_{cc,the}$(MPa)	Relative Error	$\varepsilon_{cu,the}$	Relative Error
FC1	40.56	0.0514	39.8	−0.019	0.0533	0.037	38.29	−0.056	0.0311	−0.395
PC0.2	38.07	0.0529	36.47	−0.042	0.0493	−0.068	43.59	0.145	0.0442	−0.164
NUC1.2	49.5	0.0444	46.47	−0.061	0.0612	0.378	49.13	−0.007	0.0529	0.191
FC2	51.24	0.0757	54.51	0.064	0.0709	−0.063	48.42	−0.055	0.0442	−0.416
NUC1.3	58.89	0.0738	52.22	−0.113	0.0681	−0.077	57.45	−0.024	0.066	−0.106
CPH30	32.03	0.029	32.83	0.025	0.0301	0.038	32.32	0.009	0.0218	−0.248
CPH45	27.4	0.0268	28.50	0.040	0.0294	0.097	29.26	0.068	0.0218	−0.187
CPH65	24.71	0.0084	24.23	−0.019	0.0277	2.298	26.74	0.082	0.0218	1.595

**Table 13:** Comparison of statistical measures.

	Proposed Model		FIB Model Code [42]		Wang et al. [34]	
	Confined Stress	Confined Strain	Confined Stress	Confined Strain	Confined Stress	Confined Strain
R^2^	0.983	0.905	0.817	0.576	0.846	0.152
Adjusted R^2^	0.971	0.867	0.757	0.434	0.795	0.131
RMSE	1.497	0.0067	3.674	0.008	3.026	0.018
MAE	1.006	0.006	1.758	0.001	0.276	0.012
SI	0.037	0.0012	0.077	0.141	0.064	0.301
a-20	1	1	0.8	0.800	0.8	0

## Data Availability

The original contributions presented in this study are included in the article. Further inquiries can be directed to the corresponding author.
